# Endodontic Treatment of a Maxillary First Premolar with Type IV Buccal Root Canal: A Case Report

**Published:** 2009-01-07

**Authors:** Bahareh Dadresanfar, Zohreh Khalilak, Solaleh Shahmirzadi

**Affiliations:** 1 *Department of Endodontics, Dental School, Islamic Azad University/Member of Iranian Center for Endodontic Research, Tehran, Iran*; 2 *Dental Student, Tehran, Iran*

**Keywords:** Bicuspid, Maxilla, Root Anatomy, Type IV Canals

## Abstract

The maxillary first premolar may present large number of anatomic variations. The clinician should be aware of the configuration of the pulp system. Maxillary first premolars usually have two canals. The incidence of three canals in these teeth is quite rare. This case report presents the diagnosis and clinical management of a maxillary first premolar with two distinct canals in the apical third of buccal root (type IV), drawing particular attention to tactile examination of all the canal walls.

## INTRODUCTION

Inadequate instrumentation will often lead to endodontic failure; additional root canals may be missed in cases with anatomic variations (1). The maxillary first premolar may present with a large number of anatomic variations; one possible variation is the presence of an extra canal. This is an additional challenge that should be addressed initially during case assessment as well as all the operative stages, from the access cavity design to the obturation of the root canal system (2). The reported frequency of three root canals in maxillary premolars varies from 0.5- 6% (3-6), generally with one canal in each of three roots (7). According to Weine's classification a canal that leaves the pulp chamber and divides short of the apex into two separate apical foramina, is called type IV canal (8). In Vertucci's classification of root canal configurations this is called type V (9).

Thorough literature search only found one study discussing type IV Weine's configuration in the buccal root of maxillary first premolar (10). Matuella *et al.* examined 39 buccal roots of maxillary first premolar with longitudinal sulcus. A staggering 34.3% of cases had type IV canal configuration in their buccal root (10). This case report describes the successful diagnosis and treatment of maxillary first premolar with a type IV Weine's canal configuration in buccal root.

## CASE REPORT

**Figure 1:**
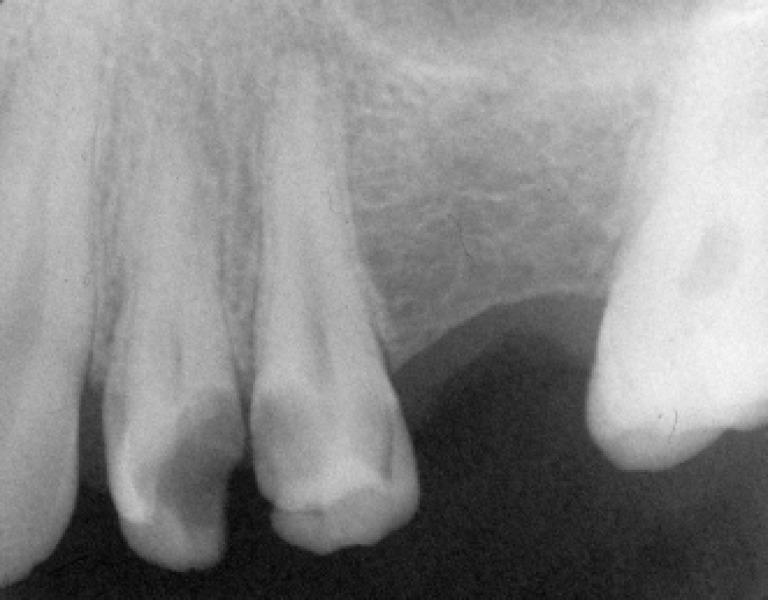
Preoperative radio-graph, showing caries and very vague two separate roots in first premolar tooth

A 24-year-old male with a non-contributory medical history sought treatment at the Dental School of Islamic Azad University. The chief complaint was "pain on chewing". Clinical examination showed a large carious lesion with pulp exposure. The tooth was not sensitive to cold testing with Endo frost (Roeko, Langenau, Germany) or electronic pulp testing (Vitality Scanner, Analytic technology, Glendora, CA, USA). Investigations for swelling, sinus tract and periodontal involvement were negative; the pulp was diagnosed as necrotic. Preoperative radiographs revealed no periapical involvement of the periodontal ligament space and very vague outline of two separate roots (Figure 1).

To ease rubber dam placement, anesthesia was obtained using Persocaine-E (DarouPakhsh Co, Tehran, Iran). A rubber dam was placed and an access cavity was prepared. In the floor of pulp chamber only two orifices were detected. Even with the exploration of the access cavity, no other orifices were found. Using two K-file size #15 (Dentsply, Maillefer, Switzerland) the working length was determined radiographic-ally. This radiograph revealed two canals with a vague outline for the buccal root.

**Figure 2:**
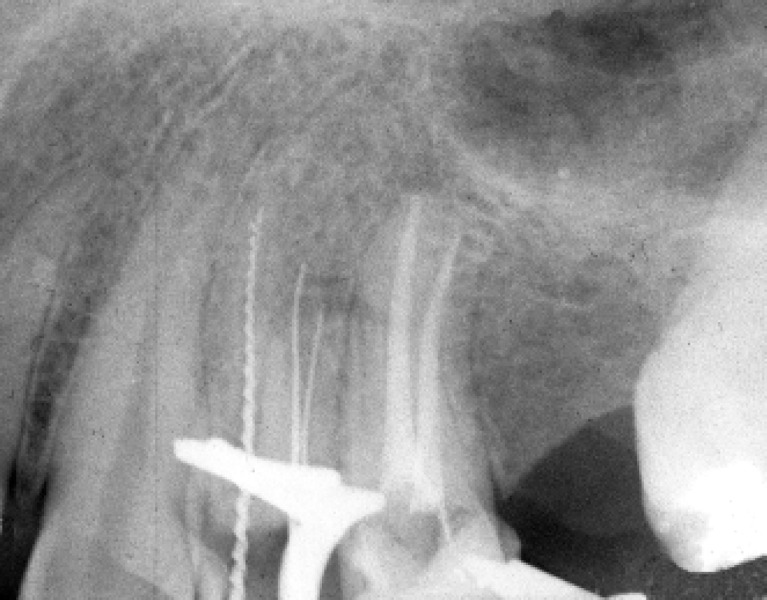
Initial radiograph, revealing two canals with a vague outline for the buccal root, three canals were negotiated

**Figure 3:**
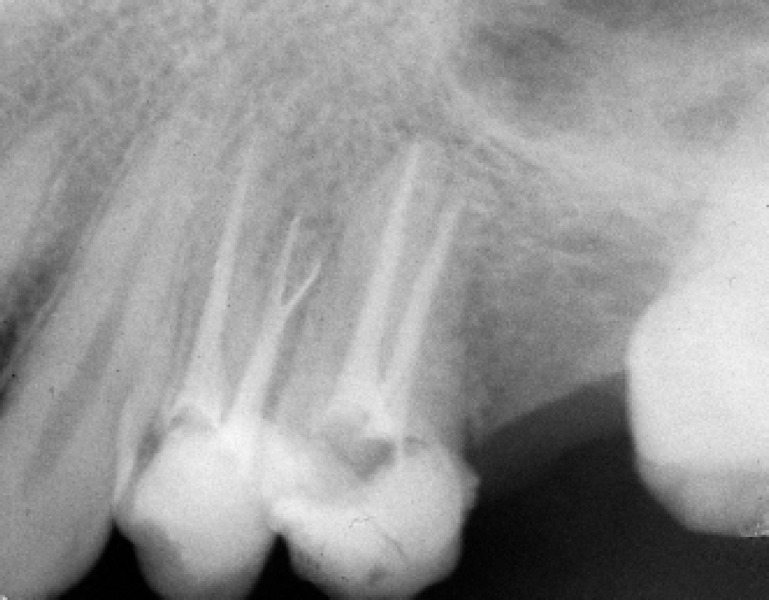
Postoperative radio-graph following the obturation of the one palatal and two buccal canals

A #15 file with severe precurve in the apical third was placed alongside to the file in the buccal canal and a radiograph was taken. This radiograph confirmed the division of buccal canals into two separate roots in the apical third (Figure 2). Then biomechanical preparation was carried out with 5.25% NaOCl as the irrigant. Master apical files #25 in the buccal canals and #30 in the palatal canal were selected, subsequently, obturation was carried out with gutta-percha and AH26 sealer (Dentsply, DeTrey, Konstanz, Germany) using lateral condensation technique (Figure 3).

## DISCUSSION

Identifying and accessing all root canals is particularly challenging in the endodontic treatment of teeth with atypical canal configuration. The maxillary first premolar has a highly variable canal and root morphology, frequently with two separate canals and two foramina (72%) (4). In treatment of three-rooted maxillary first premolars, Balleri *et al.* (11) suggested a T-shaped access outline. After thorough exploration no other orifices were found within the pulp chamber floor save the one buccal and one palatal canal. The crucial step in finding the additional buccal canal was tactile examination of all major buccal walls with a small, precurved K-file tip. After locating the canals, special attention should be paid to root canal preparation, so the obturation and hermetic seal of the canals would be possible. It is probable that if this canal had not been instrumented and obturated, a successful result may not have been achieved. This case reminds us to be vigilant when treating maxillary first premolars.

## CONCLUSION

Clinician should be aware of variations related to canal configuration and type in maxillary first premolars. Tactile sensation and inspection of canal walls with small precurved files to recognize and locate unsuspected canals is extremely important.
